# A Decade-Long Commitment to Antimicrobial Resistance Surveillance in Portugal

**DOI:** 10.3389/fmicb.2016.01650

**Published:** 2016-10-31

**Authors:** Catarina M. Marinho, Tiago Santos, Alexandre Gonçalves, Patrícia Poeta, Gilberto Igrejas

**Affiliations:** ^1^Department of Genetics and Biotechnology, School of Life and Environment Sciences, University of Trás-os-Montes and Alto DouroVila Real, Portugal; ^2^Functional Genomics and Proteomics Unit, University of Trás-os-Montes and Alto DouroVila Real, Portugal; ^3^Veterinary Science Department, University of Trás-os-Montes and Alto DouroVila Real, Portugal; ^4^UCIBIO-REQUIMTE, Faculty of Science and Technology, University Nova of LisbonLisbon, Portugal

**Keywords:** antimicrobial resistance, surveillance, molecular microbiology, enterococci, *Escherichia coli*, wildlife

## Abstract

Antimicrobial resistance (AMR) is a worldwide problem with serious health and economic repercussions. Since the 1940s, underuse, overuse, and misuse of antibiotics have had a significant environmental downside. Large amounts of antibiotics not fully metabolized after use in human and veterinary medicine, and other applications, are annually released into the environment. The result has been the development and dissemination of antibiotic-resistant bacteria due to many years of selective pressure. Surveillance of AMR provides important information that helps in monitoring and understanding how resistance mechanisms develop and disseminate within different environments. Surveillance data is needed to inform clinical therapy decisions, to guide policy proposals, and to assess the impact of action plans to fight AMR. The Functional Genomics and Proteomics Unit, based at the University of Trás-os-Montes and Alto Douro in Vila Real, Portugal, has recently completed 10 years of research surveying AMR in bacteria, mainly commensal indicator bacteria such as enterococci and *Escherichia coli* from the microbiota of different animals. Samples from more than 75 different sources have been accessed, from humans to food-producing animals, pets, and wild animals. The typical microbiological workflow involved phenotypic studies followed by molecular approaches. Throughout the decade, 4,017 samples were collected and over 5,000 bacterial isolates obtained. High levels of AMR to several antimicrobial classes have been reported, including to β-lactams, glycopeptides, tetracyclines, aminoglycosides, sulphonamides, and quinolones. Multi-resistant strains, some relevant to human and veterinary medicine like extended-spectrum β-lactamase-producing *E. coli* and vancomycin-resistant enterococci, have been repeatedly isolated even in non-synanthropic animal species. Of particular relevance are reports of AMR bacteria in wildlife from natural reserves and endangered species. Future work awaits as this threatening yet unsolved problem persists.

**GRAPHICAL ABSTRACT:**
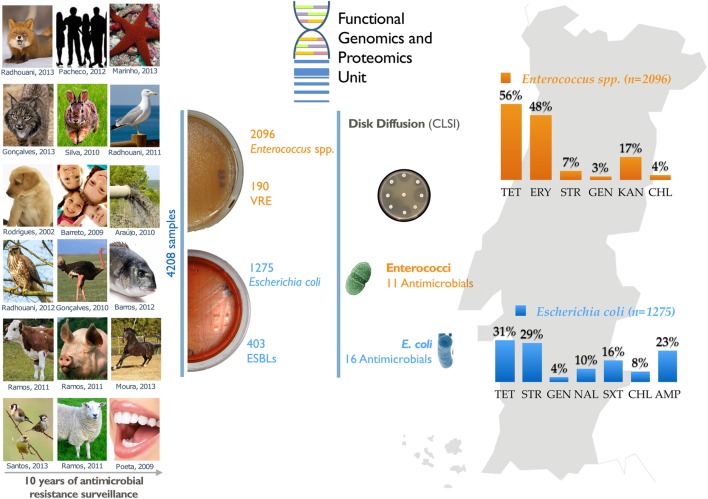
**Summary diagram of the antimicrobial resistance surveillance work developed by the UTAD Functional Genomics and Proteomics Unit**.

**Summary diagram of the antimicrobial resistance surveillance work developed by the UTAD Functional Genomics and Proteomics Unit**.

## Introduction

The early XX century witnessed the beginning of the modern antibiotic era with the first true antibiotic treatment, an asfernamina (Salvarsan) discovered by Paul Ehrlich that was successfully used to treat Syphilis (Elsner, 1910; Aminov, 2010). The reign of antibiotics in clinical practice is commonly associated with the discovery of penicillin produced by the fungus *Penicillium notatum* by Fleming (2001). However, the first wide use of antibiotics was with a sulfanamide antibiotic powder carried by Second World War solders with effectiveness against a wide range of infections (Davenport, 2012). The use of antibiotics to treat bacterial infections became one of the main scientific accomplishments; leading many scientists to believe that the threat of infectious diseases had ended (Jones, 1999). However, this golden age has come to an end in recent decades as we have become aware of the evolution of antibiotic resistance in bacterial strains, particularly in pathogens developing resistance to an extensive range of antibiotics (Davies and Davies, 2010).

Antibiotics are small secondary metabolites, either naturally produced by microorganisms or chemically synthetized, to mediate competition among bacterial populations and communities (Allen et al., 2010; Cordero et al., 2012). Antibiotics are commonly found in the environment in sub-inhibitory concentrations diminishing the growth rate of competing populations rather than killing them. Natural or synthetic antibiotics can also act as signal molecules regulating expression of a large number of transcripts in different bacteria. Moreover, antimicrobial compounds might target self-regulation of growth, virulence, sporulation, motility, mutagenesis, stress response, phage induction, transformation, lateral gene transfer, intrachromosomal recombination, or biofilm formation (Goh et al., 2002; Yim et al., 2007; Baquero et al., 2013).

Currently, antibiotics available for consumption are produced either by microbial fermentation, semi- or full synthetically manufactured. Antibiotics cause bacterial death or inhibit growth by different mechanisms of action: (a) disrupting cell walls, e.g., β-lactam and glycopeptides; (b) targeting the protein synthetic machinery, e.g., macrolides, chloramphenicol, tetracycline, linezolid, and aminoglycosides; (c) affecting the synthesis of nucleic acids, e.g., fluoroquinolones and rifampin; (d) inhibiting metabolic pathways, e.g., sulphonamides and folic acid analogs; or (e) disrupting the membrane structure, e.g., polymyxins and daptomycin (Sengupta et al., 2013).

Antimicrobial resistance (AMR) contributes to the homeostasis of microbial populations and communities by modulating the effect of naturally produced antibiotics (Cordero et al., 2012; Baquero et al., 2013). Human misuse of antibiotics has unbalanced this natural genetic system of resistance (Osterblad et al., 2001; Xi et al., 2009). In some developed countries, livestock alone represent about 50–80% of antibiotics consumption; crops, pets and aquaculture collectively account for an estimated 5%, and human therapy the remainder (Cully, 2014). The use of antibiotics in human and veterinary medicine exerts a major selective pressure leading to the emergence and spread of resistance because about 30–90% of antibiotics used are not metabolized and are discarded basically unchanged (Sarmah et al., 2006). The period of time that an antibiotic takes to be degraded after release into the environment might influence how it spreads and accumulates. For instance, penicillins are easily degraded, while fluoroquinolones and tetracyclines persist for longer. Direct discharges from antibiotics industries into water eﬄuents can be shockingly high, a reality in both developed and developing countries (Li et al., 2009; Joakim Larsson, 2014). Antibiotics are also released into the environment via agricultural application of manure and sewage water as fertilizers, and leakage from waste storage facilities (Sarmah et al., 2006; Le-Minh et al., 2010; Barrett, 2012).

The high concentration of antibiotics used prophylactically by humans might have led to contamination of human waste streams and exerted selection pressure on commensal and pathogenic bacteria. It is not surprising that non-metabolized antibiotics, resistance genes and mobile genetic elements are frequently detected in wastewaters and sewage treatment plants (Pellegrini et al., 2011). Often these compounds are not removed during sewage treatment to the extent that incidences of resistance genes are used as indicators of human impact on aquatic ecosystems (Zuccato et al., 2000; Araujo et al., 2010). Although antibiotics might be considered simply as chemical pollutants, genetic mobile elements are capable of self-replication and can be transmitted horizontally even between phylogenetically distant bacteria (Gillings and Stokes, 2012). Consequently, increased concentration of antibiotics in the environment raises the diversity and the abundance of resistance genes.

The consequences of antibiotic pollution are being actively examined, and there is an imperative need for novel strategies to curtail the release of antibiotics and bacteria from human sources (Baquero et al., 2013). Furthermore, the long-term consequences of spreading antibiotics and resistance genes cannot be predicted without quantitative analysis over suitable timescales (Chee-Sanford et al., 2009). The spread of AMR into the environment occurs not only through the contamination of natural resources but also through the food chain and direct contact between human and animals.

Currently, AMR has been reported for all antibiotic classes used both in human or veterinary medicine. An association between antibiotic use and clinical AMR proliferation has also been described (Skurnik et al., 2006; Pallecchi et al., 2008). AMR is now considered a major challenge requiring urgent action in the present to prevent a worldwide catastrophe in the future.

Portugal boasts some of the most diverse fauna and flora in Europe, and in relation to its size is considered one of the 25 biodiversity hotspots of the world (Pereira et al., 2004). Its farming systems are also numerous and varied. However, Portugal is at high risk of losing this diversity. With the mission of long-term surveillance of AMR in bacteria from different sources, the Functional Genomics and Proteomics Unit, based at the University of Trás-os-Montes and Alto Douro (Vila Real, Portugal), has recently completed a decade of unceasing research. Over the years, we have investigated the resistomes of several bacteria, mainly commensal indicator bacteria such as enterococci and *Escherichia coli* sampled from the microbiota of different animals or from other sources. Here, we present an overview of the resistance profiles of these bacteria in relation to where they were found.

## AMR as a Public Health Concern

Antimicrobial resistance has become recognized as a major clinical and public health problem (Gillings, 2013). Every year about 25,000 patients perish in the European Union (EU) because of hospital-acquired resistant bacterial infection (ECDC, 2011). Likewise, in the United States of America (USA), infections caused by antibiotic resistant bacteria are more common among the population than cancer, with at least 2 million people infected every year, at least 23,000 of whom die (CDC, 2013). Besides the clinical consequences of AMR, there are also large societal costs. The number of new antibiotics coming to the market over the past three decades has dramatically declined as several pharmaceutical companies stopped searching for antibiotics in favor of other antimicrobial drugs (Spellberg et al., 2004; Walsh, 2013). When antimicrobial drugs are discovered and developed, it is imperative to understand and predict how resistance mechanisms might evolve in order to find a method to control dissemination (Walsh, 2013). Genome-scale research may offer insight into unknown mechanisms of AMR (Tenover, 2006; Rossolini and Thaller, 2010). This will then inform decisions on appropriate policies, surveillance, and control strategies. The ideal AMR surveillance system should be able to track long-term antibiotic resistance trends, regularly alert healthcare professionals to novel resistance profiles, identify emerging resistance patterns, and create an easy-access database for physicians and scientists (Jones, 1999). To avoid a crisis, governments must recognize the importance of this valuable resource and implement a wiser and more careful use of antibiotics, raising everyone’s awareness of this issue.

Investigating the zoonotic AMR problem in its full complexity requires the collection of many types of data. Identifying the most efficient points at which intervention can control AMR mainly depends on quality collaboration between all the stakeholders involved (Wegener, 2012). The One Health concept is a universal strategy for improving connections between stakeholders in all aspects of health for humans, animals and the environment. The aim is that the synergism achieved between interdisciplinary collaborations will push forward healthcare, accelerate biomedical research breakthroughs, increase the effectiveness of public health, extend scientific knowledge and improve clinical care. When properly implemented, millions of lives can be protected and saved in the near future (Pappaioanou and Spencer, 2008). The One Health initiative is therefore an ideal framework for addressing zoonotic transmission of AMR bacteria by monitoring and managing agricultural activities, food safety professionals, human and animal clinicians, environment and wildlife experts, while maintaining a global vision of the problem.

## Resistome

The antibiotic resistome defines the pool of genes that contribute to an antibiotic resistance phenotype (D’Costa et al., 2006). Bacterial populations have exquisite mechanisms to transfer antibiotic resistance genes by horizontal transfer, mainly in densely populated microbial ecosystems. The human gut, for instance, offers ample opportunities for bacteria of different provenance to share genetic material, including the many antibiotic resistance genes harbored by gut microflora (van Schaik, 2015).

Currently, there is urgent focus on the natural resistome. A resistome database was set up in Liu and Pop (2009) and contains information on about 20,000 genes reported so far. Conclusions from several metagenomics approaches are that those 20,000 genes are just a small portion of all resistomes, since other genes with different purposes not directly related to antibiotic resistance may be implicated (Gillings, 2013). Recently, a new bioinformatics platform has been developed, the comprehensive antibiotic research database (CARD), which integrates molecular and sequence data allowing a fast identification of putative antibiotic resistance genes in newly annotated genome sequences (McArthur et al., 2013). Environmental and human associated microbial communities have been shown to harbor distinct resistomes, suggesting that antibiotic resistance functions are mainly constrained by ecology (Gibson et al., 2015). However, to date, studies investigating the roles of antibiotics and AMR outside of the clinical environment are scarce. To know more about resistome composition and the dynamics among AMR genes, new studies should be performed on AMR communities in the environment.

## AMR Surveillance in Indicator Bacteria

Living organisms are defined by the genes they possess, while the control of expression of this gene set, both temporally and in response to the environment, determines whether an organism can survive changing conditions and compete for the resources it needs to reproduce. Changes to a bacterial genome are likely to threaten the microbe’s ability to survive, but acquisition of new genes may enhance its chances of thriving by allowing growth in a formerly hostile environment. If a pathogenic bacteria gains resistance genes it is more likely to survive in the presence of antimicrobials that would otherwise eradicate it, thus compromising clinical treatments. Bacterial genomes are dynamic entities evolving through several processes, including intrachromosome genetic rearrangements, gene duplication, gene loss and gene gain by lateral gene transfer, and several other chemical and genetic factors can trigger changes by locally altering nucleotide sequences (Lawrence, 2005). AMR strains emerge and spread as a combined result of intensive antibiotics use and bacterial genetic transfer, and the incidences of resistant bacteria from diverse habitats have been increasing (Coque et al., 2008).

Recently, several pathogens have been reported as being multidrug-resistant (MDR) and believed to be ‘unbeatable’ (Grundmann et al., 2006). Incidences of MDR strains of *Staphylococcus aureus*, *E. coli*, *Salmonella*, and *Enterococcus* are of international concern and have been reported thoroughly by global health organizations (ECDC, 2013; WHO, 2014). Methicillin-resistant *Staphylococcus aureus* (MRSA) is one of the main causes of AMR associated clinical infections. Resistant isolates are still being reported at a high rate, as in Portugal where MRSA is recovered from more than 50% of isolates from humans (ECDC, 2013). In 2011, the percentage of *E. coli* isolates resistant to third-generation cephalosporins, through production of extended-spectrum beta-lactamases (ESBLs), ranged from 3 to 36% in Europe. Over the last 4 years, MDR in *E. coli* has been reported to be on the increase (ECDC, 2013). Also on European territory, enterococci resistant to high-level aminoglycosides (25–50% of recovered isolates) and vancomycin (VRE) (less than 5% of isolates) have been reported with some countries noting an increase (ECDC, 2013). *Salmonella* are considered potential sources of zoonosis. MDR *Salmonella* strains are often recovered from human and animal isolates in Europe, with high incidence in children (ECDC, 2013).

Human and animal commensal gastrointestinal bacteria are continuously subject to diverse antimicrobial pressures (Levy and Marshall, 2004). Resistant strains may act as reservoirs of resistance genes that can easily be spread to pathogenic strains (Allen et al., 2013). The potential pathogens *E. coli* and enterococci are commonly used as indicators of the selection pressure and AMR evolution through the environment (Radhouani et al., 2014).

*Escherichia coli* are facultative anaerobic Gram-negative bacteria belonging to the *Enterobacteriaceae* family. As a commensal bacterium *E. coli* colonizes the gastrointestinal tract of humans and animals, but it is found ubiquitously in soil, plants, vegetables and water (van den Bogaard and Stobberingh, 2000). *E. coli* is a potential pathogen and the leading cause of human urinary tract infection, bacteraemia and gastroenteritis, among many other infections that require therapeutic intervention (Fluit et al., 2001; Kaper et al., 2004). *E. coli* has an outstanding capacity to acquire and transfer antibiotic resistance genes from or to other bacteria, which can later be disseminated from humans to animals and to the natural environment, and vice versa (Teuber, 1999). AMR *E. coli* strains, particularly those resistant to important antibiotics, have been increasing (Collignon, 2009).

Likewise, *Enterococcus* are commensal bacteria from the intestinal flora of humans and animals, and are frequently monitored as indicators of fecal contamination of food products (Teuber and Perreten, 2000; Ramos et al., 2012). Enterococci are facultative anaerobes capable of persisting in extreme conditions caused by temperature and pH variations, dehydration, and oxidative stress. In the last two decades they have become dangerous nosocomial pathogens, persisting in hospital environments where selective pressure from the presence of antibiotics improves their resistome (Franz et al., 2011; Arias and Murray, 2012). Enterococci can cause urinary tract, wound and intra-abdominal infections, endocarditis, and bacteraemia. *Enterococcus faecalis* and *Enterococcus faecium* are the major species responsible for enterococci infections. Enterococci are intrinsically resistant to some antibiotics (β-lactams and aminoglycosides) and some species have specific AMR, such as *E. faecalis* to lincosamides and streptogramins A, and *Enterococcus gallinarum* and *E. casseliflavus* to vancomycin. They can also easily exchange resistance genes with other bacteria. Use of avoparcin, a glycopeptide antibiotic chemically similar to vancomycin, as an animal feed additive in Europe, and of high amounts of vancomycin in USA hospitals has led to the spread of VRE in animals, humans, food, and the environment (Kirst et al., 1998; Wegener, 1998). However, banning avoparcin in 2006 has not been sufficient to eradicate the AMR mechanisms present in bacteria and some VRE still persist in animals and the environment (Poeta et al., 2007b; Araujo et al., 2010; Ramos et al., 2012).

## Compilation of UTAD AMR Surveillance Data

Antibiotic prescribing practices in the EU differ widely, and the introduction of standardization would be one way to monitor use more closely and possibly reduce the emergence of resistant bacteria. The antimicrobials market has been increasing with an average annual growth of 6.6% between 2005 and 2011. Worldwide demand for antibiotics is conjectured to reach about €34.1 billion in 2016. Currently, the market is dominated by aminoglycosides, which account for 79% of demand, while penicillins account for 8%, erythromycin 7%, tetracyclines 4%, and chloramphenicol 1%. Market expansion is expected to slow down to 4.6% in coming years (GR & DS, 2012). Between 2000 and 2010, consumption of antibiotic drugs increased by 36% (from 54 billion standard units to 73 billion standard units). The greatest increase was in low- and middle-income countries, but in general, high-income countries still use more antibiotics per capita (Van Boeckel et al., 2014). Portugal is in the top 10 European countries with the greatest consumption of antibiotics, at around 21–25 doses per 1,000 habitants each year (Hede, 2014). Nonetheless, exposure to antibiotics it is not only of antibiotic consumption through prescribed human treatments but also by their use in animal production. The antibiotic environmental contamination can contribute further to the increased emergence of resistance in pathogenic and environmental bacteria. The antibiotics used in animal production may be excreted directly into the environment, or accumulate in manure which could later be spread on land as fertilizer (Almeida et al., 2014). The global total consumption of antibiotics in livestock was estimated in 2010 to be around 63,151 tons and it is project to rise 67% by 2030. Increase driven by the growth in consumer demand for livestock products in middle-income countries and a shift to large-scale farms where antimicrobials are used routinely (Van Boeckel et al., 2014).

The Portuguese annual amount of antibiotics used in human and animal consumption was discussed in a report with data from 2010 to 2011. A first comparison of the antibiotic usage in human and veterinary medicine in Portugal indicates that two-thirds of the consumed antibiotics are used in veterinary medicine whereas the rest one-third in human medicine. For human medicine antibiotic prescription increased 4.5 tons (81.4–85.9 tons). In these two registered years, the annual amount of the different antibiotic groups was markedly larger for penicillins, which alone accounted for more than 65.0%, followed by quinolones (13.0%), macrolides (7.0%), cephalosporins (6.0%), and sulfonamides (5.0%). In veterinary medicine the annual amount of antibiotics sold was 179 tons in 2010 and 163 tons in 2011. Tetracyclines and penicillins were the most sold therapeutic solutions for the both years (Almeida et al., 2014).

**FIGURE 1:**
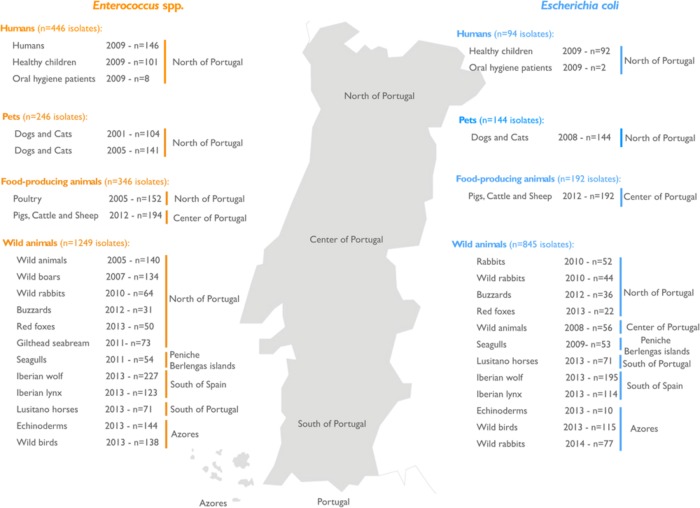
**Geographical distribution in Portugal showing the sources of samples containing bacterial isolates**. The number and provenance of samples containing enterococci (orange) and *Escherichia coli* (blue) are shown. Both types of bacteria were frequently isolated from several samples from humans, pets, food-producing animals, and wild animals.

Although it has been registered a decrease in the use of antibiotics in Portugal, their consumption and use is still high. Surveying AMR trends among bacteria is necessary to inform risk analyses and guide public policy. Over one decade at University of Trás-os-Montes and Alto Douro (UTAD), the antimicrobial profiles of indicator bacteria *E. coli* (**Table 1**) and enterococci (**Table 2**) have been studied in samples isolated from more than 75 different sources in Portugal. **Figure 1** summarizes the data according to the four groups of sources from which samples were collected: humans, pets, food-producing animals, and wild animals.

**Table 1:** Description of all sources of *Escherichia coli* isolates analyzed in a decade of antimicrobial resistance (AMR) surveillance in Portugal by the UTAD Functional Genomics and Proteomics Unit.

Sampling group	Sample source	Recovered samples (*n* = 1,841	Recovered isolates (*n* = 1,275)	Reference
Pets	Dogs and cats	75	144	Costa et al., 2008
Humans	Children	118	92	Barreto et al., 2009
	Oral hygiene patients	46	2	Poeta et al., 2009
Food-producing animals	Pigs, cattle, and sheep	198	192	Ramos et al., 2012
Wild animals	Diarrheic rabbits	52	52	Poeta et al., 2010
	Wild rabbits	77	44	Silva et al., 2010
	Buzzards	42	36	Radhouani et al., 2012
	Red foxes	52	22	Radhouani et al., 2013
	Several wild animals	72	56	Costa et al., 2006
	Seagulls	53	53	Radhouani et al., 2009
	Lusitano horses	90	71	Moura et al., 2010
	Iberian wolf	237	195	Goncalves et al., 2013a
	Iberian lynx	27	18	Goncalves et al., 2012
	Iberian lynx	98	96	Goncalves et al., 2013b
	Echinoderms	250	10	Marinho et al., 2013
	Wild birds	218	115	Santos et al., 2013
	Wild rabbits	136	77	Silva et al., 2010

**Table 2:** Description of all sources of *Enterococcus* spp. isolates analyzed in a decade of AMR surveillance in Portugal by the UTAD Functional Genomics and Proteomics Unit.

Sampling group	Sample source	Recovered samples (*n* = 2,730)	Recovered isolates (*n* = 2,287)	Reference
Pets	Dogs and cats	104	104	Rodrigues et al., 2001
	Dogs and cats	220	142	Poeta et al., 2005a
Humans	Humans	220	146	Poeta et al., 2005a
	Children	118	101	Barreto et al., 2009
	Oral hygiene patients	46	8	Poeta et al., 2009
Food-producing animals	Poultry	220	152	Poeta et al., 2005a
	Pigs, cattle, and sheep	198	194	Ramos et al., 2012
Wild animals	Wild animals	77	140	Poeta et al., 2005b
	Wild boars	67	134	Poeta et al., 2007b; Silva et al., 2010
	Wild rabbit	77	64	Silva et al., 2010
	Gilthead seabream	118	73	Barros et al., 2011
	Seagulls	57	54	Radhouani et al., 2011
	Buzzards	42	31	Radhouani et al., 2012
	Iberian wolf	237	227	Goncalves et al., 2013a
	Iberian lynx	30	27	Goncalves et al., 2011
	Iberian lynx	98	96	Goncalves et al., 2013b
	Echinoderms	250	144	Marinho et al., 2013
	Lusitano horses	90	71	Moura et al., 2010
	Red foxes	52	50	Radhouani et al., 2013; Santos et al., 2013
	Wild birds	218	138	Santos et al., 2013

The fecal samples used in all studies, either from Human or animal origin, were obtained following similar guidelines. The fecal samples (one per animal) were recovered from patients (in the case of human studies) and directly from the intestinal tract in the animal studies. Samples were transported in Cary-Blair medium from the place of recovery to the Centre of Studies of Animal and Veterinary Sciences (CECAV) facilities for processing. For *E. coli* selection, fecal samples were seeded onto Levine agar plates and incubated for 24 h at 37°C. One colony per sample with typical *E. coli* morphology was selected and identified by classical biochemical methods (Gram-staining, catalase, oxidase, indol, Methyl-Red, Voges-Proskauer, citrate, urease, and triple sugar iron), and by the API 20E system (BioMerieux, La Balme Les Grottes, France). As for the enterococcal isolation, fecal samples were sampled onto Slanetz-Bartley agar plates and incubated at 37°C for 24 h. One colony with typical enterococcal morphology were identified to the genus level by cultural characteristics, Gram staining, catalase test, and bile-aesculin reaction. Antibiotic susceptibility tests were made by the disk diffusion method following CLSI recommendations. 16 antibiotics were tested against *E. coli* isolates: ampicillin (10 mg), amoxicillin + clavulanic acid (20 mg + 10 mg), cefoxitin (30 mg), cefotaxime (30 mg), ceftazidime (30 mg), aztreonam (30 mg), imipenem (10 mg), gentamicin (10 mg), amikacin (30 mg), tobramycin (10 mg), streptomycin (10 mg), nalidixic acid (30 mg), ciprofloxacin (5 mg), sulfamethoxazole-trimethoprim (1.25 mg + 23.75 mg), tetracycline (30 mg), and chloramphenicol (30 mg). The identification of phylogenetic groups was performed as described by Clermont *E. coli* phylo-typing method (Clermont et al., 2000). In the case of enterococci, the isolates were tested for 11 antibiotics: [vancomycin (30 μg), teicoplanin (30 μg), Ampicillin (10 μg), streptomycin (300 μg), gentamicin (120 μg), kanamycin (120 μg), chloramphenicol (30 μg), tetracycline (30 μg), erythromycin (15 μg), quinupristin-dalfopristin (15 μg), and ciprofloxacin (5 μg)], also by the disk diffusion method. Species identification was confirmed by polymerase chain reaction (PCR) using primers and conditions for the different enterococcal species. *E. coli* and enterococci isolates with resistance to one or more antibiotics were selected for the characterization of antibiotic-resistance genes. Their DNA was extracted and tested by PCR with specific primers already published. *E. coli* resistant isolates were screened for the following resistance genes: *bla*TEM and *bla*SHV (in ampicillin-resistant isolates); *tet*A and *tet*B (in tetracycline resistant isolates); *aad*A, *aad*A5, *str*A, and *str*B (in streptomycin-resistant isolates); and *sul*1, *sul*2, and *sul*3 (in sulfa-methoxazoletrimethoprim-resistant isolates). Also, the presence of *int*I, *int*I2 and *qac*ED + *sul*1 genes was analyzed by PCR in all sulfamethoxazole-trimethoprim-resistant isolates. On the other hand, enterococci resistant isolates were tested by PCR for detection of the following resistance genes: *erm*(B) and *erm*(C) (in erythromycin-resistant isolates), *tet*(M) and *tet*(L) (in tetracycline-resistant isolates), *aph*(3′)-IIIA (in kanamycin-resistant isolates), *aac*(6′)-aph(2^′′^) (in gentamicin-resistant isolates), *ant*(6)-Ia (in streptomycin-resistant isolates), *vat*(D) and *vat*(E) (in quinupristin/dalfopristin-resistant isolates), and *van*(A) (in vancomycin-resistant isolates). Positive and negative controls were used in all PCR reactions, from the strain collection of the University of Trás-os-Montes and Alto Douro (Portugal).

In this collation of data, a total of 1,841 samples were collected and screened for *E. coli*. The overall *E. coli* recovery rate was 69.26% (**Table 1**), but rates were different among the different sampling groups, ranging from 96.97% in fecal samples from food-producing animals down to 52.08% in fecal samples from pets. These are higher percentages than the *E. coli* recovery rate (51%) from humans, domestic and food-producing animal feces, or sewage and surface water in the USA (Sayah et al., 2005). A total of 2,539 samples were screened for enterococci, and a total of 2,096 enterococci isolates were obtained giving a recovery rate of 78.1% (**Table 2**). Rates of recovery from the four sampling groups were similar (75.93% for pet fecal samples, 66% for human samples, 82.78% for fecal samples from food-producing animals; and 88.39% for fecal samples from wild animals). These rates are very similar to previously reported rates (77%) of enterococci recovery from human, animal and environmental samples in several European countries (Kuhn et al., 2003).

## *Escherichia coli* Data Description

**FIGURE 2:**
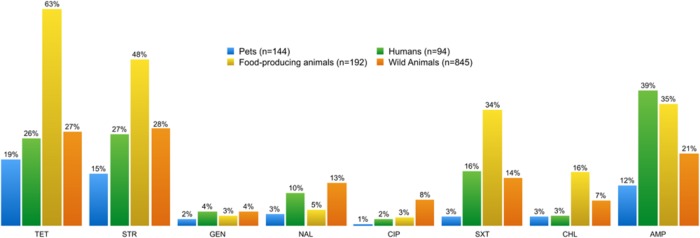
**Percentage of phenotypic antibiotic resistance detected in enterococci and *E. coli* isolates from each of the four sampling groups**. The resistance profile of enterococci from food-producing animals is distinct from those of the other sampling groups, showing the highest resistance to tetracycline, streptomycin, trimethoprim-sulfamethoxazole, and chloramphenicol. Enterococci from pets displayed the lowest resistance profile to all tested antibiotics. TET, tetracycline; STR, streptomycin; GEN, gentamicin; NAL, nalidixic acid; CIP, ciproflaxin; SXT, trimethoprim-sulfamethoxazole; CHL, chloramphenicol; AMP, ampicillin.

**FIGURE 3:**
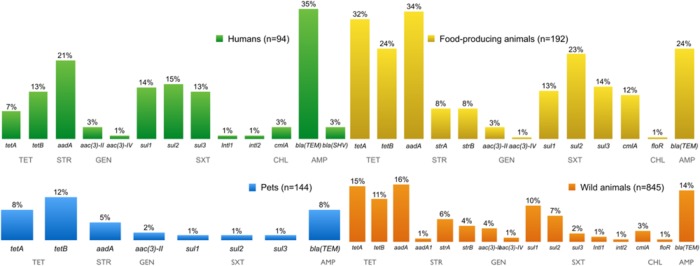
**Percentage of antimicrobial resistance (AMR) genes detected in *E. coli* resistant isolates from all sources over a decade**.

Phenotypic and genotypic studies were used to assess the AMR profile of the isolates. **Figures 2** and **3** summarize the results of tests for antibiotic resistance and identification of associated resistance genes in *E. coli* isolates, comparing the four different sample groups described in **Table 1**. Resistance to all the antibiotics tested was found among the samples. Remarkably, *E. coli* from food-producing animals showed higher rates of resistance to tetracycline, streptomycin, trimethoprim-sulfamethoxazole, and chloramphenicol than *E. coli* from humans (**Figure 2**). Indeed fecal *E. coli* isolates recovered from food-producing animals all across Europe have shown a high prevalence of resistance to these antimicrobial agents (Guerra et al., 2003; Enne et al., 2008; Lapierre et al., 2008). Despite the ban on chloramphenicol use in farming in Europe since 1994, food-producing animals still carry resistant strains. Regular and multipurpose use of antibiotics, dissemination of resistant bacteria via fecal contamination and intensive animal production have turned husbandry facilities into important reservoirs of AMR bacteria (van den Bogaard and Stobberingh, 2000). High levels of resistance to tetracycline and streptomycin have been found in bacteria from wild animals. The high prevalence of tetracycline resistance in either *E. coli* or enterococci found in different wild animal species might be related to the recurrent use of this antibiotic in veterinary medicine. The same may be true of the prevalence of resistance to erythromycin, ampicillin, aminoglycosides, and quinolones. One of the main routes of transmission of resistant strains and resistant genes has been shown to be through the food chain (Aarestrup et al., 2008). Antibiotics can be administered to animals to treat (therapy) or prevent (prophylaxis or metaphylaxis) illness and in the past were also used to promote animal growth. Several classes of antimicrobial agents are frequently used in veterinary medicine. A 2013 report showed that in 2011 in 25 countries from the EU/European Economic Area, tetracyclines (37%) were the most sold antimicrobial class for administering to food-producing species, followed by penicillins (23%), sulfonamides (11%), macrolides (8%), lincosamides (2.9%), aminoglycosides (2%), trimethoprim (1.6%), and fluoroquinolones (1.6%) (European Medicines Agency, 2013). Antimicrobial use in veterinary medicine and AMR in commensal *E. coli* from various food-producing animals has indeed been correlated (Chantziaras et al., 2014). Ampicillin is a β-lactam used as a farm animal growth promoter (Sayah et al., 2005). *E. coli* from human samples differ from *E. coli* from other sampling groups in terms of their ampicillin resistance, which is of particular importance considering that ampicillin is a first-line antibiotic commonly effective against a broad spectrum of pathogens.

Comparing AMR *E. coli* from independent sources, several genes conferring AMR were found in resistant isolates (**Figure 3**). Fourteen different *tet* genes have been reported in Gram-negative bacteria, encoding proteins active in the three mechanisms of tetracycline resistance, namely an eﬄux pump, ribosomal protection, or direct enzymatic inactivation of the antibiotic. The resistance genes *tet*A and *tet*B confer resistance to tetracycline, and were found at similar frequencies in all different sources of resistant *E. coli*. However, *tet*A and *tet*B are more likely to be associated with tetracycline resistance in *E. coli* isolated from human and animal samples (Bryan et al., 2004).

The streptomycin resistance genes *aad*A, *str*A, *str*B, and *aad*A1 were found, in descending order of frequency, in resistant *E. coli*. The *str*A and *str*B genes encode streptomycin-inactivating enzymes which confer streptomycin resistance in many Gram-negative bacteria distributed worldwide. The *aad*A gene encodes an adenylylation enzyme that modifies streptomycin and, in the *aad*A1 gene cassette, is often found in streptomycin-resistant *E. coli* strains from human, animal, and food samples (Saenz et al., 2004).

Gentamicin resistance genes *aac*(3)-II and *aac*(3)-IV were present in resistant *E. coli* isolates. Trimethoprim-sulfamethoxazole-resistant *E. coli* isolates had *sul*1, *sul*2 and/or *sul*3 genes, and the integrons *intI*1 and *intI*2 genes, encoding class 1 and class 2 integrases, respectively. The *sul1*, *sul2*, and *sul3* genes each encode dihydropteroate synthases to confer plasmid-mediated resistance to sulphonamides (Perreten and Boerlin, 2003; Skold, 2010). The *sul1* gene is frequently found linked to other resistance genes in class 1 integrons, while *sul2* is normally located on small plasmids of the *incQ* incompatibility group, or other small plasmids represented by pBP1 (Skold, 2010). The *sul1* and *sul2* genes have been regularly found at similar frequencies among sulphonamide resistant Gram-negative clinical isolates. However, an increase in prevalence of the *sul2* gene has been observed. The association between *sul2* and multiresistance plasmids, which may be co-selected through the use of other antimicrobial agents, might explain the increased prevalence (Blahna et al., 2006; Frank et al., 2007).

**FIGURE 4:**
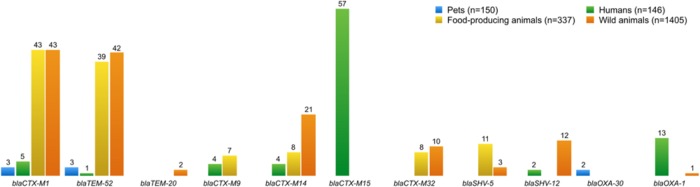
**Number of ESBL-producing *E. coli* and resistance genes present in all isolates from different groups**. blaCTX-M15 was the most frequent ESBL gene recovered from *E. coli*, followed by blaCTX-M-1 and blaTEM-52. Otherwise, blaTEM-20, blaOXA-30, and blaCTX-M-9 genes were found in a few ESBL-producing *E. coli*.

Chloramphenicol resistant *E. coli* isolates have the resistance genes *cml*A and *flo*R, both coding eﬄux pumps that reduce the intracellular concentration of antibiotics by exporting different molecules (Saenz et al., 2004). Some infections are difficult to treat because of bacterial expression of multidrug eﬄux systems causing resistance. ESBLs confer resistance to penicillins and most cephalosporins, including third-generation cephalosporins, and more than 700 different ESBLs have now been described (van Duijn et al., 2011). A total of 403 isolates of ESBL-producing *E. coli* were obtained from all four sampling groups: humans (*n* = 138), pets (*n* = 8), food-producing animals (*n* = 116), and wild animals (*n* = 141). **Figure 4** shows all genes encoding ESBLs that were detected in these isolates. The most prevalent ESBL genes were *bla*_CTX-M-1_ (*n* = 94) and *bla*_TEM-52_ (*n* = 85), specifically isolated from *E. coli* from food-producing and wild animals, followed by the *bla*_CTX-M-32_ (*n* = 57) gene found in isolates from human samples. The mechanisms of resistance against one antibiotic might not be exclusive, and activity may be exhibited against other similar compounds. For instance, it has been proven that mutations in the genes encoding penicillin-inactivating enzymes (e.g., TEM and SHV) can also confer resistance to third-generation cephalosporins or β-lactamase inhibitors (Martinez et al., 2007).

## Enterococci Data Description

**FIGURE 5:**
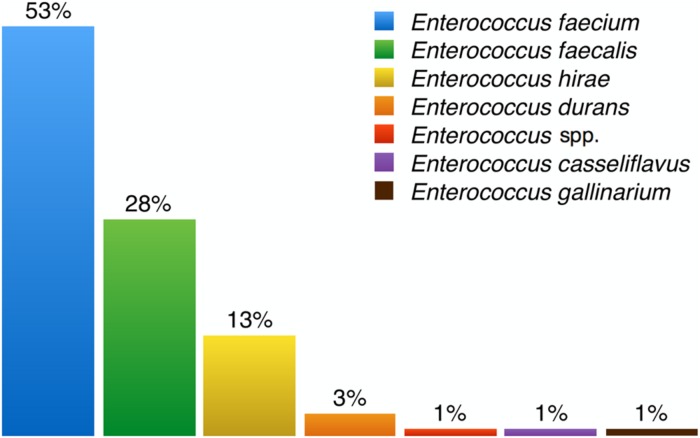
**Percentages of enterococci species isolated from samples**. *Enterococcus faecium* is the most frequently isolated enterococci species among all recovered samples and *Enterococcus gallinarum* the least.

Several species of enterococci were recovered from different environments, with some being more common than others depending on the source of the sample. **Figure 5** summarizes all data on enterococci species prevalence reported in the publications listed in **Table 2**. *E. faecalis* is the most common enterococcal species in human stools and the most frequent cause of clinical infection. The natural resistance of this species to penicillin, cephalosporins, and quinolones has contributed to its emergence as a cause of nosocomial infection. However, *E. faecium* is inherently more antibiotic-resistant and has also gradually become a major hospital pathogen (French, 2010; Radimersky et al., 2010). Overall from all the enterococci species we isolated, *E. faecium* was by far the most frequently recovered (52.9%), followed by *E. faecalis*, *E. hirae*, *E. durans*, *E. casseliflavus*, and *E. gallinarum* (**Figure 5**).

**FIGURE 6:**
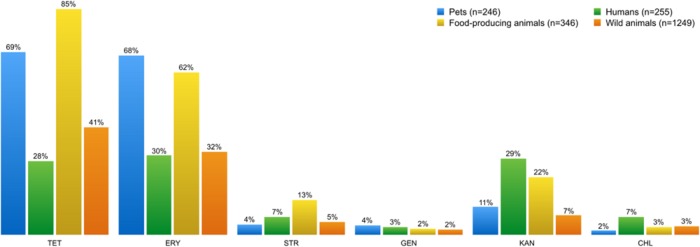
**Percentage of phenotypic antibiotic resistance detected in *Enterococcus* spp. isolates from each of the four sampling groups**. Enterococci isolates showed high resistance levels to tetracycline and erythromycin, regardless of the source. However, food-producing animals and pets stand out from the other sampling groups. Streptomycin, kanamycin, and chloramphenicol-resistant enterococci are more frequent in samples from humans than from other sources. TET, tetracycline; ERY, erythromycin; STR, streptomycin; GEN, gentamicin, KAN, kanamycin; CHL, chloramphenicol.

**FIGURE 7:**
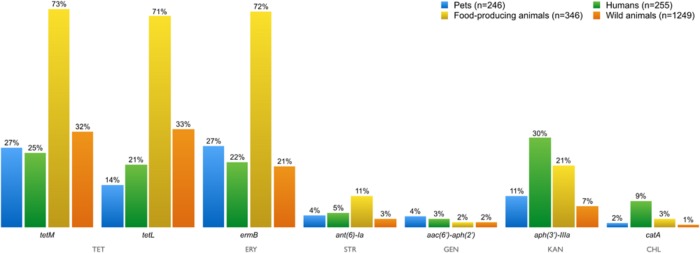
**Percentage of AMR genes detected in resistant Enterococci isolates from all sources over a decade**. Resistance genes tet(M)/tet(L), erm(B), ant(6)-Ia, aac(6′)-aph(2′), aph(3′)-IIIA, and catA were present in enterococci isolates resistant to tetracycline, erythromycin, streptomycin, gentamicin, kanamycin, and chloramphenicol, respectively. TET, tetracycline; ERY, erythromycin; STR, streptomycin; GEN, gentamicin; KAN, kanamycin, CHL, chloramphenicol.

**Figures 6** and **7** show the collated antibiotic resistance data of enterococci isolates from the different sources listed in **Table 2**. Remarkably, more enterococci from the group of food-producing animals were resistant to tetracycline and erythromycin than from the human group (**Figure 6**). Many enterococci resistant to tetracycline and erythromycin were also found in the wild animals group. Enterococci resistant to kanamycin and chloramphenicol were more frequent in samples from humans than from other sources.

The distribution of AMR genes in the different sampling groups is illustrated in **Figure 7**. The *tet*M and *tet*L genes are associated with the tetracycline-resistance phenotype in enterococci. Tetracycline resistance due to TetL eﬄux proteins has been becoming more frequently reported among Gram-positive bacteria from animal sources (Figueiredo et al., 2009; Radhouani et al., 2010).

Erythromycin-resistance in enterococci is usually associated with the presence of the *erm*B gene. The *erm*B and *tet*M genes have been frequently identified together in the same isolate, probably because of they both occur in the mobile conjugative transposon *Tn1545*, prevalent in clinically important Gram-positive bacteria (De Leener et al., 2004). The resistance genes *ant*(6)-Ia, *aac*(6′)-*aph*(2′), *aph*(3′)-IIIA and *cat*A were present in enterococci isolates resistant to streptomycin, gentamicin, kanamycin, and chloramphenicol, respectively. Resistance to all tested antibiotics has been showed in isolates from all groups.

**FIGURE 8:**
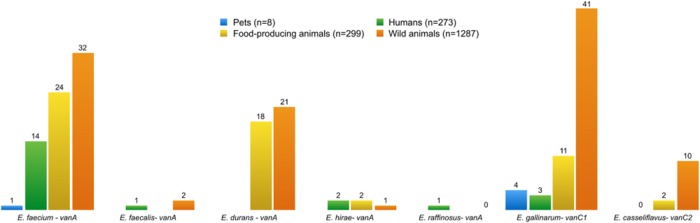
**Number of vancomycin-resistant enterococci species, and resistance genes present in all isolates from different groups**. *E. gallinarum* and *E. casseliflavus* are intrinsically resistant to vancomycin, and harbor the genes vanC1 and vanC2, respectively, in their genome.

The presence of VRE isolates was noted in all four groups (**Figure 8**): humans (*n* = 21), pets (*n* = 5), food-producing animals (*n* = 57), and wild animals (*n* = 107). The presence of VRE isolates in diverse samples from Portugal is likely to be related to the use of avoparcin to promote growth of food animals in Europe since the 1970s until its prohibition in 2006. VRE was first characterized in Uttley et al. (1988), and has been repeatedly isolated since then from several sources. Many antimicrobials used in food animals belong to the same classes as those used in humans, leading to concerns about cross-resistance (Furuya and Lowy, 2006). For instance, vancomycin is still the most widely used antibiotic to treat serious infections like MRSA. Vancomycin resistance has emerged in *S. aureus* through acquisition of the *vanA* gene from VRE (Chang et al., 2003). It is important therefore to highlight the higher number of vancomycin resistant determinants found in wild animals compared to humans. In Europe, spread from animals to humans appears to have occurred outside hospital facilities, which explains why VRE is more commonly found in the environment than in the community (Furuya and Lowy, 2006).

## Conclusion

Generalized use of antibiotics by humans is a recent addition to the natural and ancient process of antibiotic production and resistance evolution that still occurs on a global scale in the soil (Woolhouse et al., 2015). The main attributed cause of the rise and spread of AMR is the extensive clinical use of antibiotics in both human and veterinary medicine (Levy, 2001). However, antibiotic use in veterinary medicine alone does not explain the spread and persistence of AMR bacteria in animal populations in which the antibiotic use is discontinued or else is very sporadic or limited like in wildlife. AMR does not have borders, so it can freely cross through several populations, and homologous resistance genes have been reported in bacteria from pathogens, normal flora and soil. Farm animals are a significant factor in this system and they are still exposed to large amounts of antibiotics and act as reservoirs of many resistance genes. Other risk factors such as antibiotic residues in the environment and AMR gene dissemination in different human and animal populations also have an impact on the AMR levels (Alali et al., 2009). We are currently facing the potential loss of antimicrobial therapy, so it is essential to continue tracking AMR.

## Future Perspectives

Human use of antibiotics for medicine and agriculture may have consequences far beyond their intended applications. The environment is a major reservoir of resistant organisms and antibiotic drugs freely circulating; though not much is known about the antibiotic resistome, shading their hypothetical impact on clinically important bacteria (Allen et al., 2010). Several resistance genes have been reported in distinct bacterial species, from diverse sources (Poeta et al., 2007a; Pallecchi et al., 2008; Gillings and Stokes, 2012). Nonetheless, more studies using standardized methods are required to recognize roles and patterns of antibiotic resistance genes in microbial communities. In the meantime, it is important to moderate the use of antimicrobials and restrain the rise and dissemination of AMR bacteria, so the management of emerging zoonotic diseases and their impacts might be forecasted (Allen et al., 2010). The foreseen decline in antibiotics effectiveness and the current lack of new antimicrobials on the horizon to replace those that become ineffective brings added urgency to the protection of the efficacy of existing drugs (Carlet, 2012; WHO, 2014). Meanwhile, omics approaches to generate molecular data on AMR mechanisms will continue to be the foundation of our work at the Functional Genomics and Proteomics Unit at UTAD.

## Author Contributions

CM and TS wrote the manuscript. AG, PP, and GI helped interpret compiled data. GI conceived the review. All the authors reviewed and contributed to the manuscript.

## Conflict of Interest Statement

The authors declare that the research was conducted in the absence of any commercial or financial relationships that could be construed as a potential conflict of interest.
